# Active Surveillance in Early Thyroid Cancer: A Meta-Analysis

**DOI:** 10.3390/diagnostics14232628

**Published:** 2024-11-22

**Authors:** Li-Jen Liao, Yukiko Ono, Shun-Fa Hung, Yong-Chen Chen, Wan-Lun Hsu

**Affiliations:** 1 Department of Otolaryngology, Far Eastern Memorial Hospital, New Taipei City 220, Taiwan; dtent87@gmail.com; 2 Head and Neck Cancer Surveillance and Research Study Group, Far Eastern Memorial Hospital, New Taipei City 220, Taiwan; 3 Department of Electrical Engineering, Yuan Ze University, Taoyuan City 320, Taiwan; 4 Data Science Center, College of Medicine, Fu Jen Catholic University, New Taipei City 242, Taiwan; yulya.o.18.xd@gmail.com (Y.O.); yongchenchen0824@gmail.com (Y.-C.C.); 5 Graduate Institute of Business Administration, College of Management, Fu Jen Catholic University, New Taipei City 242, Taiwan; 6 Evidence-Based Medicine Center, Far Eastern Memorial Hospital, New Taipei City 320, Taiwan; shunfa@mail.femh.org.tw; 7 Post-Baccalaureate Program in Nursing, College of Medicine, Fu Jen Catholic University, New Taipei City 242, Taiwan; 8 Master Program of Big Data in Medical Healthcare Industry, College of Medicine, Fu Jen Catholic University, New Taipei City 242, Taiwan

**Keywords:** active surveillance, early thyroid cancer, meta-analysis

## Abstract

**Background/Objectives**: There remain several challenges to the acceptance and standardization of active surveillance (AS) in patients with early thyroid cancer. The purpose of this study was to update the evidence of tumor progression outcomes in AS to support resolution of clinical concerns and to examine the effect of follow-up duration, period context such as diagnostic techniques, and tumor size at baseline on outcomes. **Methods**: PubMed, Web of Science, and Embase were used to search for original articles in English until January 2024. The main outcomes were the pooled proportion of patients who showed tumor growth ≥ 3 mm, metastasis to cervical lymph nodes, and conversion to delayed thyroid surgery. A single-arm meta-analysis was performed using a random-effects model. **Results**: Fourteen studies with 7291 patients were included in the analysis. Pooled results showed that 5.6% (95% CI: 4.2–7.4) of patients increased tumor diameter by more than 3 mm, 1.1% (95% CI: 0.9–1.4) developed cervical lymph node metastases by clinical diagnosis and 3.6% (95% CI: 3.1–4.2) by pathology, 12.7% (95% CI: 9.9–16.1) of patients converted to delayed surgery, with 31.6% (95% CI: 25.3–38.7) of those conversions being due to tumor progression. Subgroup analysis showed a small difference in the proportion of outcomes by baseline tumor size but no increase in the proportion of tumor progression or conversion to surgery due to other factors. **Conclusions**: This meta-analysis suggests long-term stability in the proportion of tumor progression in AS and less susceptibility to external influences.

## 1. Introduction

The reported incidence of thyroid cancer has been rapidly increasing worldwide due to advancements in and widespread use of diagnostic techniques [1,2,3]. This upward trend consists mainly of an increase in papillary carcinomas, and a survey in the United States showed that approximately half of the newly diagnosed cases involved tumors with a maximum diameter of 1 cm or less, and nearly 90% involved tumors with a maximum diameter of 2 cm or less [2]. The favorable prognosis of most of these small thyroid cancers, coupled with concerns about the risks associated with persistent hypoparathyroidism and recurrent laryngeal nerve palsy from surgical treatment [4,5,6], has made overdiagnosis and overtreatment a problem in recent years.

Active surveillance (AS) of thyroid cancer, first introduced in Japan, is a minimally invasive treatment strategy aims to reduce the risks and costs associated with surgery while maintaining the patient’s quality of life [5,7]. In AS, cancer dynamics are closely monitored through periodic ultrasound and blood tests for papillary thyroid microcarcinoma (PTMC) with a maximum diameter of 1 cm or less which has no risk factors such as lymph node metastasis, invasion of surrounding tissues, distant metastasis, or proximity to the trachea or recurrent nerve. Disease progression in AS is mostly defined by an increase in tumor diameter of more than 3 mm or the occurrence of new lymph node metastasis, and delayed thyroid surgery is performed according to disease progression and patients’ changing preferences. The effectiveness and safety of AS have been presented in many studies [8,9], making AS currently the standard treatment option in Japan [10], and the American Thyroid Association endorsed AS as a treatment strategy based on individualized and careful evaluation in 2015 [11].

On the other hand, although the acceptance of AS in clinical settings is gradually progressing, it is still limited to a few countries and institutions. This is due to issues such as evidence of long-term safety and the availability of diagnostic techniques that allow accurate assessment of the primary tumor and lymph node status. Additionally, several studies have discussed expanding the definition of target tumor size, which also poses a challenge in standardizing treatment. The decision to retain tumor requires very careful deliberation for both patients and clinicians, necessitating more detailed supporting evidence. Hence, this study aims to address these concerns by updating the evidence on outcomes for early thyroid cancer patients under AS and examining the effect of follow-up period, study initiation period, and baseline tumor size on these outcomes.

## 2. Materials and Methods

PubMed, Web of Science, and Embase (from inception to January 2024) were systematically searched for articles in English using the following search expressions: (“active surveillance” OR “AS”) AND (“thyroid cancer” OR “PTC” OR “thyroid carcinoma” OR “thyroid microcarcinoma” OR “thyroid micro-carcinoma” OR “thyroid micro-carcinoma” OR “PTMC” OR “thyroid nodules”). References to relevant literature were also reviewed for pertinent publications. Two authors (Ono Y and Hsu WL) independently evaluated the titles and abstracts of the retrieved literature, and full-text details were obtained for those deemed relevant. Any discrepancies were resolved by a senior investigator (Liao LJ). The inclusion criteria were as follows: (1) study design must be a clinical trial, cohort study, case-control study, or cross-sectional study; (2) the study must involve early stage thyroid cancer less than 2 cm (cT1N0M0); (3) the study must manage thyroid cancer by AS with follow-up every 6 to 12 months as an alternative to immediate surgery; and (4) the study must measure one or more of the following indicators to assess disease progression: percentage of patients showing an increase in the maximum tumor diameter, cervical lymph node metastasis, and conversion to delayed thyroid surgery. Studies that focused on populations of children under 18 years of age studies that were difficult to analyze due to insufficient data were excluded. In cases of duplicate study samples, the study with the larger sample size was included, and the study with the smaller sample size was excluded.

We extracted key study data using a standardized form that included study participant characteristics and study design; for studies with cohorts with different intervention methods other than the AS cohort, data were extracted for the AS cohort only. In addition to AS patient outcomes, the data obtained included study demographics such as first author, year of publication, country, time direction of data collection, baseline period, sample size, age, gender, thyroid cancer size definition, maximum tumor diameter at baseline, tumor pathology definition, and follow-up duration.

The main outcomes were defined as an increase in primary tumor (≥3 mm increase in maximum diameter from baseline), metastasis to cervical lymph nodes, and conversion to delayed thyroid surgery. Of these, metastasis to cervical lymph nodes was evaluated separately for cervical lymph node metastasis based on the clinical diagnosis during AS and for that based on the pathological diagnosis after delayed surgery. For delayed thyroid surgery, the following patients were evaluated: “all patients who underwent AS and were converted to delayed surgery” and “patients who were converted to delayed surgery because of tumor progression”. The definition of tumor progression for the surgical decision encompassed increased tumor diameter and metastasis to cervical lymph nodes, as well as extrathyroidal invasion of the tumor and increased tumor volume.

A single group meta-analysis was performed using a random effects model, pooling the proportion of each outcome (95% CI). Results calculated by the fixed effects model were presented as a sensitivity analysis. Heterogeneity across studies was assessed using Cochran Q (heterogeneity χ2) and *I*^2^ statistics. For the main analysis, studies were grouped by outcome and analyzed. Subgroup analyses were performed by tumor diameter definition in the AS selection, median (or mean) follow-up period, and baseline start period. For subgroup analyses, we required a minimum of three independent studies to justify the analyses. For the sensitivity analysis, as well as the use of a fixed-effects model, studies that included non-papillary cancers in the study population and studies that included patients who underwent AS for less than one year were excluded, respectively, and then analyzed as in the main analysis to verify the consistency of the results. The analysis was performed using R version 4.3.3 with the “metafor” and “meta” packages.

## 3. Results

### 3.1. Study Flow and Characteristics

**Figure 1:**
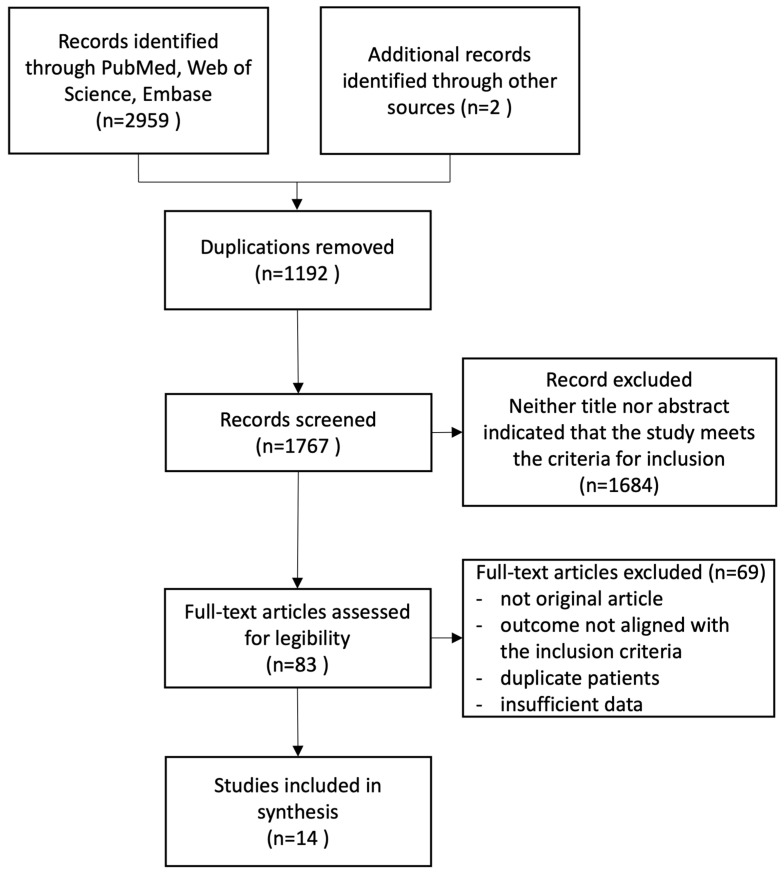
Study selection flowchart.

A total of 2961 articles were identified, and after initial screening, 83 were reviewed in full text (Figure 1). The full-text review resulted in the exclusion of 69 articles due to not original article, outcome not aligned with the inclusion criteria, duplicate study coverage, or insufficient data, ultimately resulting in the inclusion of 14 studies and 7291 patients in the analysis.

Of the 14 total studies, all used a nonrandomized design, with data collected prospectively in 13 studies and retrospectively in one study (Table 1 and Table 2). The earliest study by baseline start date was 1993, and the most recent study was 2017. Four studies had a median or mean follow-up of less than 2 years, four studies from 2 to 3 years and 4 months, and six studies from more than 3.5 years. Six of the 14 studies excluded patients who received AS less than one year from the start of the study for analysis. The study with the least number of participants had 41, the study with the most had 3222, and there were 4 of the 14 studies that included more than 500 participants. Geographically, three studies each were from Japan and China, two each from Korea and the United States, and one each from Canada, Italy, Argentina, and Colombia. The study participants consisted of 3854 from Japan, 1379 from China, 1076 from Korea, 595 from the United States, 151 from Canada, 93 from Italy, 41 from Argentina, and 102 from Colombia. The age distribution of each study was concentrated between 40 and 60 years, and women accounted for 63–88% of participants in the studies. The majority of the identified studies involved papillary thyroid tumors, and only three included non-papillary tumors. Regarding the indication criteria for AS, half of the studies defined the tumor diameter as 10 mm or less, four studies defined it as 14 to 15 mm or less, three studies defined it as 20 mm or less, and there was variability among the studies in the indication criteria that exceeded 10 mm. Furthermore, eight studies reported including multifocal tumors, but only one study provided data grouped by unifocal versus multifocal.

Information on initial treatment is presented in Table 3. The criteria for conversion from AS to initial treatment were described in 13 studies. The majority of studies adopted an increase in tumor diameter ≥ 3 mm as a criterion for recommending initial treatment, while four studies used different criteria for tumor size increase [12,13,14,15], with a more permissive criterion of ≥5 mm in the study by Ho et al. [13]. Three studies also used tumor volume increase as a conversion criterion [13,15,16]. In all studies collected, conversion to surgery was recommended as initial treatment. The operations performed mainly included unilateral lobectomy or total thyroidectomy and central or lateral lymph node dissection. Five studies reported on the proportion of thyroid operations actually performed, of which four studies showed that unilateral lobectomy exceeded the proportion of total thyroidectomy. For lymph node dissection, the proportion of prophylactic versus therapeutic dissection was reported in three studies, of which in two study prophylactic central lymph node dissection was performed in all DTS patients.

**Table 1:** Characteristics of included studies.

Author	Country	Data Collection	Baseline Period	No. of Patients	Age (Year)	Sex (Female, %)
Sawka, 2024 [17]	Canada	Prospective	2016–NR	151	55 (46–68) ^§^	78
Miyauchi, 2023 [18]	Japan	Prospective	1993–2019	3222	57.0 (20.0–92.0) ^§^	88
Zhuge, 2023 [19]	China	Prospective	2014–2021	779	≤40: 42.0% 41–50: 34.2% >50: 23.9%	81
Liu, 2023 [16]	China	Prospective	2017–2022	485	43 (16–78)	78
Liu, 2022 [20]	China	Prospective	2013–NR	115	41.8 ± 10.3 *	86
Lee, 2022 [12]	Korea	Prospective	2016–2020	706	49.3 ± 11.8 *	63
Tuttle, 2022 [21]	USA	Prospective	NR	483	52 ± 15 *	77
Ho, 2022 [13]	USA	Prospective	2014–2021	112	49.1 (38.2–60.4) ^§^	73
Nagaoka, 2021 [22]	Japan	Prospective	1995–2016	571	53.1 ± 12.7 *	87
Molinaro, 2020 [23]	Italy	Prospective	2014–2018	93	44 ± 15 *	77
Smulever, 2020 [14]	Argentina	Prospective	2014–NR	41	<60: 87.8% ≥60: 12.2%	88
Sanabria, 2020 [15]	Colombia	Prospective	2015–NR	102	50.6 ± 16.3 *	83
Sakai, 2019 [24]	Japan	Prospective	1995–2016	61	54.4 ± 10.7 *	77
Oh, 2018 [25]	Korea	Retrospective	2002–2017	370	51.0 ± 11.7 *	77

* Mean ± SD, ^§^ Median (IQR).

**Table 2:** Characteristics of early thyroid cancer and active surveillance.

Author	Size Definition (mm)	Maximum Tumor Diameter (mm)	Histological Type	Follow-Up (Year)
Sawka, 2024 [17]	≤10	7 (2–10) ^§^	Papillary carcinoma	2016–NR *
Miyauchi, 2023 [18]	<20	11 (8–13) ^§^	Papillary carcinoma	1993–2019
Zhuge, 2023 [19]	≤10	≤6: 73.0% >6: 27.0%	Highly suspicious thyroid nodules	2014–2021
Liu, 2023 [16]	≤10	0.5 (0.2–1.0) ^§^	Highly suspicious thyroid nodules	2017–2022
Liu, 2022 [20]	≤10	4 (3–6) ^§^	Papillary carcinoma	2013–NR
Lee, 2022 [12]	≤10	6.2 ± 1.6	Papillary carcinoma	2016–2020
Tuttle, 2022 [21]	≤15	≤10: 75% >10 ≤15: 25%	Papillary carcinoma	NR
Ho, 2022 [13]	≤20	11 (9–15) ^§^	Papillary carcinoma	2014–2021
Nagaoka, 2021 [22]	≤10	NR	Papillary carcinoma	1995–2016
Molinaro, 2020 [23]	≤14	9.4 ± 2.5 *	Papillary carcinoma	2014–2018
Smulever, 2020 [14]	≤15	≤10: 69% >10, ≤15: 31%	Papillary carcinoma	2014–NR
Sanabria, 2020 [15]	≤15	10.3 ± 5.8 *	Carcinoma	2015–NR
Sakai, 2019 [24]	10–20	11.7 ± 1.1 *	Papillary carcinoma	1995–2016
Oh, 2018 [25]	≤10	5.9 ± 1.7 *	Papillary carcinoma	2002–2017

* Mean ± SD, ^§^ Median (IQR). NR: not reported.

**Table 3:** Characteristics of initial treatment.

Author	Conversion Criteria for Initial Treatment	Type of Initial Treatment Performed	Type of Lymph Node Dissection Performed *
Sawka, 2024 [17]	Details reported	Lobectomy: 7 (64%) Total thyroidectomy: 2 (18%) Isthmectomy: 1 (9%) Radiofrequency ablation: 1 (9%)	CND: 2 (20%)
Miyauchi, 2023 [18]	Details reported	Lobectomy: 206 (52%) Total thyroidectomy: 188 (47.7%)	CND + LND: 44 (11%) CND: 350 (89%)
Zhuge, 2023 [19]	Details reported	NR	NR
Liu, 2023 [16]	Details reported	Lobectomy: 30 (67%) Endoscopic + lobectomy: 1 (2%) Total thyroidectomy: 14 (31%)	CND 45 (100%)
Liu, 2022 [20]	Reported	NR	NR
Lee, 2022 [12]	Details reported	NR	NR
Tuttle, 2022 [21]	NR	NR	NR
Ho, 2022 [13]	Details reported	NR	NR
Nagaoka, 2021 [22]	Details reported	Insufficient data	Insufficient data
Molinaro, 2020 [23]	Details reported	Insufficient data	Insufficient data
Smulever, 2020 [14]	Details reported	Lobectomy/Total thyroidectomy	Insufficient data
Sanabria, 2020 [15]	Details reported	Lobectomy: 5 (38%) Total thyroidectomy: 8 (62%)	pCND: 1 (8%) tCND: 1 (8%) LND: 1 (8%)
Sakai, 2019 [24]	Details reported	Mainly lobectomy	pCND: 11 (100%) SLND: 2 (18%)
Oh, 2018 [25]	Details reported	Lobectomy: 41 (71%) Total thyroidectomy: 17 (29%)	pCND: 58 (100%)

* Denominator for percentage is total number of patients who underwent delayed thyroid surgery. NR: not reported, DTS: delayed thyroid surgery, CND: central neck dissection, LND: lateral neck dissection, pCND: prophylactic central neck dissection, tCND: therapeutic central neck dissection.

### 3.2. Increase in Maximum Tumor Diameter ≥ 3 mm

**Figure 2:**
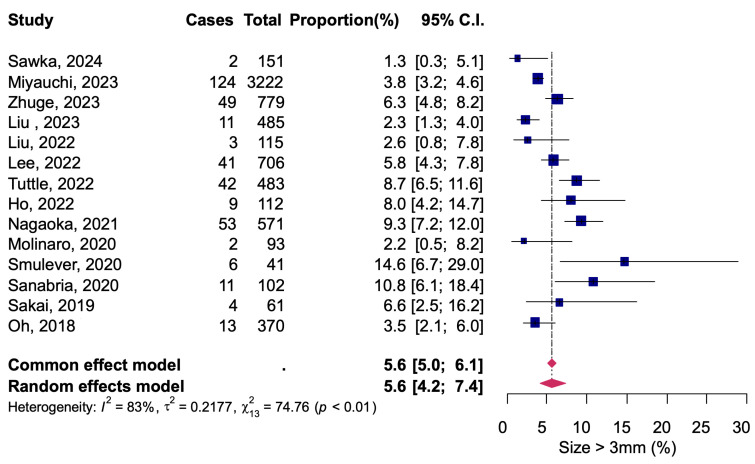
Tumor size increase by diameter during active surveillance [12,13,14,15,16,17,18,19,20,21,22,23,24,25].

All studies evaluated the increase in diameter of early-stage thyroid tumors during AS, with a total of 7291 patients, of whom 370 patients were observed to have a diameter increase of ≥3 mm compared to baseline. The pooled proportion by meta-analysis was 5.6% (95% CI: 4.2–7.4), and there was significant heterogeneity among the studies (*I*^2^ = 83%, Q = 74.76, *p* < 0.01) (Figure 2).

### 3.3. Metastasis to Cervical Lymph Nodes

**Figure 3:**
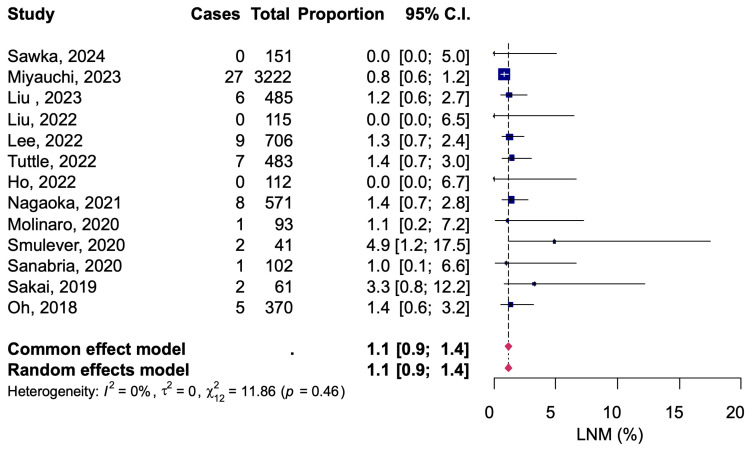
Clinical metastatic to cervical lymph node during active surveillance [12,13,14,15,16,17,18,20,21,22,23,24,25].

**Figure 4:**
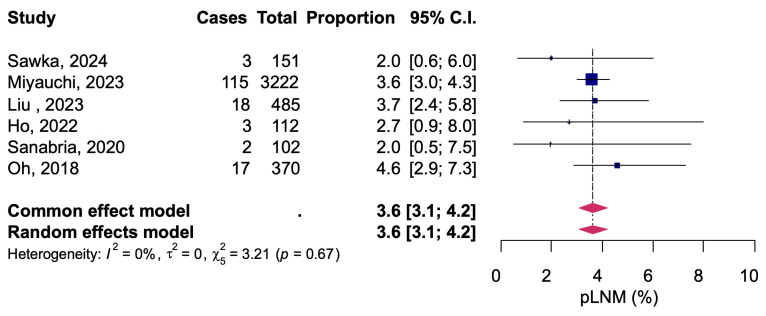
Pathological metastatic to cervical lymph node during active surveillance [13,15,16,17,18,25].

All studies evaluated cervical lymph node metastases, but one study reported only a combined number of patients with new thyroid nodal detection and was excluded from the analysis. Of a total of 6512 patients, 68 had cervical lymph node metastases detected by clinical examination during AS, for a pooled proportion of 1.1% (95% CI: 0.9–1.4) (Figure 3). There was no significant heterogeneity among the studies (*I*^2^ = 0%, Q =11.86, *p* = 0.46). The results of pathological diagnosis of cervical lymph node metastases were reported in 6 studies. Of a total of 4442 patients who underwent AS, 158 patients had a definitive diagnosis of cervical lymph node metastasis at pathology tests that were performed with delayed surgery. The pooled proportion was 3.6% (95% CI: 3.1–4.2) with no significant heterogeneity between studies (*I*^2^ = 0%, Q = 3.21, *p* = 0.67) (Figure 4).

### 3.4. Conversion to Delayed Thyroid Surgery

**Figure 5:**
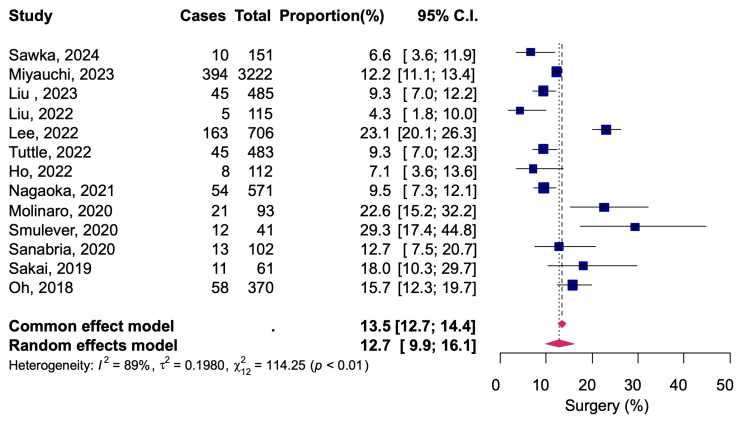
Conversion to delayed thyroid surgery during active surveillance [12,13,14,15,16,17,18,20,21,22,23,24,25].

**Figure 6:**
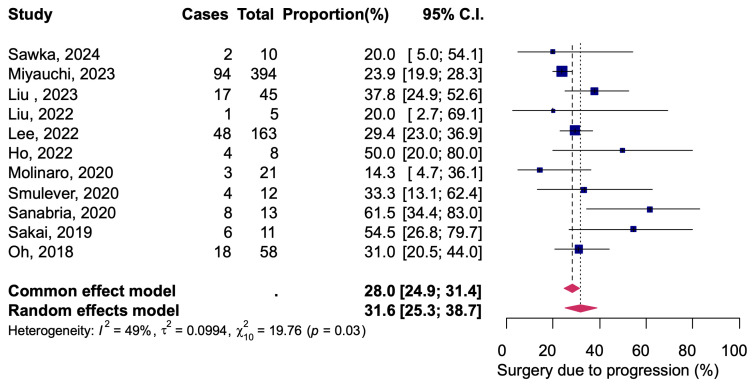
Delayed thyroid surgery due to tumor progression out of all delayed thyroid surgery [12,13,14,15,16,17,18,20,23,24,25].

Thirteen studies reported 839 of 6512 patients in total converted from AS to delayed surgery due to change of patient intention, tumor progression, or other reasons, resulting in a pooled proportion of 12.7% (95% CI: 9.9–16.1) (Figure 5). There was a high heterogeneity among the studies (*I*^2^ = 89%, Q = 114.25, *p* < 0.01). Data on the determinants of delayed surgery were presented in 11 studies. Of the patients converted to delayed surgery, the reason was tumor progression in 205 of 839 patients, for 31.6% (95% CI: 25.3–38.7) (Figure 6). Heterogeneity between studies was moderate *I*^2^ = 49%, Q = 19.76, *p* = 0.03).

### 3.5. Subgroup Analysis

Grouped by median (or mean) of follow-up duration, there was a slight increase in all outcomes in the group with 2 to 3.4 years of follow-up compared to the group with less than 2 years of follow-up (Table 4). On the other hand, when comparing the group with 2 to 3.4 years of follow-up to the group with more than 3.5 years of follow-up, the latter group had a 3.5% higher proportion of patients converted to delayed surgery due to tumor progression, while all other proportions showed a decrease. In the analysis grouping patients by baseline start period, there were no differences in results between the pre-2002 and post-2013 groups. In the analysis by baseline tumor diameter, there was a slightly higher proportion of increased maximum tumor diameter ≥ 3 mm, cervical lymph node metastasis by clinical diagnosis, and conversion to delayed thyroid surgery in the group of studies that targeted tumor ≤ 20 mm, compared to the group of studies that targeted tumor ≤ 10 mm.

### 3.6. Sensitivity Analysis

A fixed-effects model meta-analysis was calculated as a sensitivity analysis, and no serious deviations were found from the results obtained with the random-effects model in any of the analyses (Figure 2, Figure 3, Figure 4, Figure 5 and Figure 6). The results were approximately equivalent to the main analysis after excluding studies that included non-papillary carcinomas in the study population and studies that included patients who underwent AS for less than one year, respectively (Table S1).

## 4. Discussion

Our meta-analysis confirmed a consistently low proportion of patients with early thyroid cancer having increased tumor size, cervical lymph node metastases, and conversion to delayed surgery due to tumor progression. We also examined the impact of follow-up time, the background of the study period, and tumor size at baseline on outcomes during AS, and showed the stability of AS outcomes and the reliability of the treatment strategy.

In the main analysis, 5.6% of patients had increased tumor diameter ≥ 3 mm, and 1.1% of patients had a clinical diagnosis of cervical lymph node metastasis development. These results are compatible with those of previous studies [8,26]. The pooled proportion of cervical lymph node metastases detected in pathological diagnoses was 3.6%. Four of the 14 studies included in this analysis compared the proportion of lymph node metastases by pathology in patients who underwent immediate surgery versus delayed surgery, and only one study showed a statistical difference between the two groups. A previous large study of patients with PTMC reported that lymph node metastases based on postoperative pathology had no effect on tumor-free survival at 10 years postoperatively [27]. These findings suggest the safety of AS, however, further reliable evidence is needed to validate these results in the future. Larger sample sizes should be considered, along with an assessment of the proportion of lymph node dissection procedures performed and the ratio of prophylactic to therapeutic dissection in the overall patient population undergoing surgery. Additionally, investigation with continued follow-up of patients who underwent delayed surgery is necessary. Regarding to the criteria for initial treatment selection, most studies recommend thyroid lobectomy, whereas total thyroidectomy and neck dissection are recommended in cases of suspected cervical lymph node metastases. Regarding AS patients who were converted to delayed surgery, the pooled proportion was 12.7%. Of these, 31.6% were due to tumor progression, and more than half were due to change of patient preference. These results are consistent with those of previous meta-analyses [8,9]. The decision to leave the tumor, knowing the risk involved, causes anxiety and stress for patients and affects their ability to make decisions [28], though it was shown that the quality of life and psychological status of patients who underwent AS was better than that of patients who underwent immediate surgery [29,30]. Therefore, the importance of continued appropriate information provision and psychological support to patients regarding the current status of their tumors and the advantages and disadvantages of applicable treatments should be reemphasized in the decision to continue AS.

In the subgroup analysis, obvious differences in outcomes were observed only among the groups with different baseline tumor diameters. The most apparent difference was in tumor enlargement, which was 2.6% higher in the study group with tumor diameters ≤ 20 mm as a baseline definition than in the group with tumor diameters ≤ 10 mm. However, the 7.3% in the tumor diameter ≤ 20 mm group was not clinically high, and there was little difference in lymph node metastasis.

Grouped by follow-up duration, the proportion of tumor progression was slightly higher in the 2- to 3.4-year study group compared to the <2-year study group, while the ≥3.5-year study group was slightly lower when compared to the 2- to 3.4-year study group. In a previous meta-analysis, the most pronounced change in the cumulative percentage of patients with an increase in tumor diameter ≥ 3 mm during AS was between the second and fourth years from the start of AS [26], and there is a similarity between these results and our meta-analysis results. It is also notable that the proportion of lymph node metastases in our analysis showed a similar trend to the increase in tumor diameter, and three of the study groups with the longest follow-up period had an average follow-up period of more than 7 years.

In the implementation of AS, the development of a medical system for early and accurate diagnosis is an important issue for clinicians who decide to adopt AS. Since 2000, innovations in high-resolution imaging and Doppler technology in cervical ultrasound echo equipment have dramatically improved the accuracy of diagnosis of thyroid cancer and lymph node metastases. On the basis of this background, we performed a subgroup analysis by baseline start period and found little difference in any of the outcomes between the study groups started before 2002 and those started in 2013 or later. Three of the four studies in the pre-2002 group were conducted in Japan, while the post-2013 group included a total of nine studies conducted in seven countries other than Japan. These findings suggest that external factors such as advances in diagnostic technology and differences in healthcare systems in different regions may not have had a decisive impact on AS outcomes and may support the fact that AS is a widely applicable treatment strategy.

The limitations of this study are firstly the partial overlap in the distribution of stratification criteria among the different groups in the subgroup analysis. Tumor diameter and follow-up duration as stratification factors may have had a relatively large influence on our analysis, and future studies are recommended to provide more detailed information for each sub-cohort to enhance clarity and accuracy. Second, because of the lack of studies, it was not possible to analyze the effect of characteristics such as ethnicity or multifactorial thyroid cancer. Further accumulations of evidence are needed to improve the external validity of the results, especially since the distribution of countries and institutions studied is unbalanced. Third, the patients in this analysis may have included non-papillary cancer patients. This is because biopsy of thyroid nodules was not mandatory in some studies, but a sensitivity analysis that limited the study population to papillary thyroid cancer confirmed the consistency of the results. Fourth, there was variation in the treatment of patients lost to follow-up in each study, with some studies not reporting the number of patients lost to follow-up. It is possible that the results may have been under-estimated by these patients.

For future research, it is also important to monitor the long-term outcomes of patients who undergo delayed surgery in order to better understand the safety and efficacy of AS treatment strategies. At the same time, a comprehensive and detailed examination of the economic aspects of AS versus immediate surgery is essential to assess the viability of AS, given the wide variations in health care systems and cost structures in different countries.

## 5. Conclusions

It was shown that AS in patients with early thyroid cancer has a stable tumor progression outcome over a 3.5-year period of AS and has a high applicability as a treatment strategy. In addition, there was no apparent high risk of clinically problematic progression for tumors with baseline diameters > 10 mm. More detailed information regarding subgroup-specific data, criteria for conversion to initial treatment, types of delayed surgery procedures, and untraceable cases is required to assess applicability in populations with different backgrounds, such as race, nationality, and number of tumor nests, and to increase the reliability of the results.

## Figures and Tables

**Table 4:** Subgroup analysis by diameter by follow-up duration, start of baseline period, and diameter at baseline.

	Increase ≥3 mm	cLNM	pLNM	DTS	DTS Due to Progression
	PP (95% C.I.)	PP (95% C.I.)	PP (95% C.I.)	PP (95% C.I.)	PP (95% C.I.)
Follow up duration <2.0 y 2.0–3.4 y ≥3.5 y					
	4.9 (5.0–8.0)	0.8 (0.2–2.8)	2.0 (0.8–4.7)	12.9 (10.7–18.6)	29.7 (8.7–65.1)
	5.9 (3.7–9.3)	1.4 (0.8–2.5)	4.2 (2.7–6.5)	12.9 (6.2–24.8)	30.6 (25.1–36.6)
	5.5 (3.3–9.2)	1.2 (0.8–1.7)	3.6 (3.0–4.2)	10.7 (8.9–12.9)	34.1 (20.4–51.0)
Start of baseline period					
1993–2010	5.3 (3.0–9.4)	1.2 (0.8–1.9)	3.7 (3.1–4.4)	12.7 (10.3–15.7)	30.5 (20.1–43.2)
2011–2017	5.3 (3.6–7.8)	1.3 (0.8–2.0)	3.2 (2.2–4.5)	12.6 (7.9–19.5)	33.1 (24.7–42.8)
Diameter at baseline					
≤10 mm	4.7 (3.3–6.6)	1.0 (0.8–1.4)	3.7 (3.2–4.3)	12.2 (8.5–16.8)	28.1 (23.5–33.1)
≤20 mm	7.3 (4.8–11.0)	1.7 (1.0–2.8)	2.2 (1.1–4.4)	13.4 (8.8–19.9)	37.8 (22.5–56.0)

PP: pooled proportion, CI: confidence interval, cLNM: clinical lymph node metastases, pLNM: pathological lymph node metastases, DTS: delayed thyroid surgery.

## Data Availability

The data supporting the findings of this study are available from publicly accessible databases and published literature. All data sources are cited within the article, and the datasets can be accessed through the corresponding references.

## Supplementary Materials

The following supporting information can be downloaded at: https://www.mdpi.com/article/10.3390/diagnostics14232628/s1, Table S1. Sensitivity analysis.
