# Supplementary material for: Active Surveillance in Early Thyroid Cancer: A Meta-Analysis
Source: Diagnostics (Basel). 2024 Nov 22;14(23):2628. doi: 10.3390/diagnostics14232628 (PMC11640271; doi:10.3390/diagnostics14232628)
Supplement: Supplementary file 1 [file diagnostics-14-02628-s001.zip › diagnostics-3297935-Supplementary material.pdf]

## Supplementary Materials

**Table S1.** Sensitivity analysis.

| | Overall | Sensitivity analysis (1) | Sensitivity analysis (2) |
|---|---|---|---|
| | PP (95% C.I.) | PP (95% C.I.) | PP (95% C.I.) |
| Diameter > 3mm | 5.6 (4.2 - 7.4) | 5.6 (4.0 - 7.8) | 4.5 (3.0 - 6.6) |
| cLNM | 1.1 (0.9 - 1.4) | 1.2 (0.9 - 1.7) | 1.2 (0.7 - 1.9) |
| pLNM | 3.6 (3.1 - 4.2) | 3.6 (3.1 - 4.3) | 3.7 (3.0 - 4.4) |
| DTS | 12.7 (9.9 – 16.1) | 13.1 (9.9 – 17.0) | 13.8 (9.3 – 20.0) |
| DTS due to progression | 31.6 (25.3 – 38.7) | 28.1 (32.1 - 33.7) | 26.1 (22.9 - 29.7) |

Sensitivity analysis (1): Studies that included non-papillary cancers in the study population were excluded in the analysis.
Sensitivity analysis (2): Studies that included patients who underwent AS for less than one year were excluded in the analysis.
PP: pooled proportion, CI: confidence interval, cLNM: clinical lymph node metastases, pLNM: pathological lymph node metastases, DTS: delayed thyroid surgery.
